# A multi-model deep learning approach for the identification of coronary artery calcifications within 2D coronary angiography images

**DOI:** 10.1007/s11548-025-03382-5

**Published:** 2025-05-08

**Authors:** Edoardo De Rose, Ciro Benito Raggio, Ahmad Riccardo Rasheed, Pierangela Bruno, Paolo Zaffino, Salvatore De Rosa, Francesco Calimeri, Maria Francesca Spadea

**Affiliations:** 1 Department of Mathematics and Computer Science, University of Calabria, Pietro Bucci, 87036 Rende, Calabria Italy; 2 Institute of Biomedical Engineering, Karlsruhe Institute of Technology, Fritz-Haber-Weg 1, 76131 Karlsruhe, Baden-Württemberg Germany; 3 Experimental and Clinical Medicine, Magna Graecia University, Viale Europa, 88100 Catanzaro, Calabria Italy; 4 Medical and Surgical Sciences, Magna Graecia University, Viale Europa, 88100 Catanzaro, Calabria Italy; 5 DLVSystem Srl, Viale della Resistenza 19/C, 87036 Rende, Calabria Italy

**Keywords:** Coronary angiography, Deep learning, Coronary artery calcification, Clinical decision support system

## Abstract

**Purpose:**

Identifying and quantifying coronary artery calcification (CAC) is crucial for preoperative planning, as it helps to estimate both the complexity of the 2D coronary angiography (2DCA) procedure and the risk of developing intraoperative complications. Despite the relevance, the actual practice relies upon visual inspection of the 2DCA image frames by clinicians. This procedure is prone to inaccuracies due to the poor contrast and small size of the CAC; moreover, it is dependent on the physician’s experience. To address this issue, we developed a workflow to assist clinicians in identifying CAC within 2DCA using data from 44 image acquisitions across 14 patients.

**Methods:**

Our workflow consists of three stages. In the first stage, a classification backbone based on ResNet-18 is applied to guide the CAC identification by extracting relevant features from 2DCA frames. In the second stage, a U-Net decoder architecture, mirroring the encoding structure of the ResNet-18, is employed to identify the regions of interest (ROI) of the CAC. Eventually, a post-processing step refines the results to obtain the final ROI. The workflow was evaluated using a leave-out cross-validation.

**Results:**

The proposed method outperformed the comparative methods by achieving an F1-score for the classification step of 0.87 (0.77$-$0.94) (median ± quartiles), while for the CAC identification step the intersection over minimum (IoM) was 0.64 (0.46$-$0.86) (median ± quartiles).

**Conclusion:**

This is the first attempt to propose a clinical decision support system to assist the identification of CAC within 2DCA. The proposed workflow holds the potential to improve both the accuracy and efficiency of CAC quantification, with promising clinical applications. As future work, the concurrent use of multiple auxiliary tasks could be explored to further improve the segmentation performance.

**Supplementary Information:**

The online version contains supplementary material available at 10.1007/s11548-025-03382-5.

## Introduction

The identification and measurement of coronary artery calcification (CAC) has several clinical applications, particularly in cardiovascular risk stratification and pre-intervention planning [1]. Accurate knowledge of the location and amount of CAC is crucial for estimating the procedure’s risks and the complexity of the intervention [2]. Moreover, angio-based CAC detection and quantification may inform decisions regarding the need for further procedures, such as intracoronary imaging [3], thus ensuring more effective and tailored interventions. Indeed, CAC poses a significant challenge to adequate coronary dilatation, potentially leading to alternative revascularization methods such as surgical revascularization or the selection of advanced debulking strategies. Such strategies, including intracoronary lithotripsy, rotational or orbital ablation, and excimer laser procedures, are commonly available in most cardiac catheterization laboratories [4]. While 2D coronary angiography (2DCA) is the standard for visualizing the coronary vascular tree, it faces several challenges in detecting CAC. These include: motion artifacts caused by physiological movements, reduced contrast due to overlapping anatomical structures, and the difficulty in manually identifying small, often obscured calcifications without contrast agents. These limitations complicate the detection process, making it less reliable. Current clinical practice for identifying and measuring CAC relies on physicians visually inspecting 2DCA image frames. To detect CACs, physicians use contrast-enhanced frames to guide their analysis of non-contrast frames. This process is time-consuming, heavily depends on the operator’s expertise, and, in challenging cases, it may require consensus among multiple experts.

Recent studies have explored deep learning (DL)-based approaches to improve CAC detection, but most of them focus on computed tomography (CT)[5] rather than 2DCA. CT Coronary angiograms (CTCA) provide better contrast and take advantage of standardized Hounsfield unit (HU) values to train DL model to segment CACs [6]. De Vos et al.[7] were among the first to introduce a 2D CNN-based approach to evaluate the amount of CAC. However, detecting CACs in 2DCA is significantly more challenging because the modality lacks the inherent contrast and HU-based information available in CTCA. Despite their potential, CTCA is not widely adopted in interventional cardiology due to higher costs and lower compatibility with standard clinical workflows, where 2DCA remains the primary modality [8].

Some works exploited 2DCA acquisitions [9–11] to automatically segment the entire coronary tree in 2DCA using U-Net [12], transformer-based approaches, and YOLO [13].

Other authors [14–17] have addressed stenosis detection in 2DCA using two-step DL frameworks. In all these cases, the authors recognized that stenosis segmentation in 2DCA is impractical due to the inherent limitations of the imaging modality. Instead, they focused on detection, comparing different neural network architectures to optimize performance for this specific task.

For instance, Danilov et al  [16] trained and compared eight promising detectors based on different DL architectures to localize stenotic lesions on angiography image series.

In this work, we propose a multi-model DL framework for identifying CAC in 2DCA images, drawing inspiration from the methodology presented by Huo et al. [18]. To the best of our knowledge, this is the first attempt of performing such a task on projective images. Our approach mirrors the clinical workflow, by using both contrast-enhanced and non-contrast frames to guide decisions and it was validated on a dataset of 44 acquisitions from 14 patients. The contribution of this paper can be summarized as follows:

- We propose a method to automatically synchronize frames with and without contrast, in order to match the anatomy in different phases of the heartbeat;
- We propose a multi-model deep learning architecture (ResNet integrated with a U-Net-like decoder) to identify the regions of interest (ROIs) where CAC is present, in order to support the clinicians’ decision-making.

**Fig. 1:**
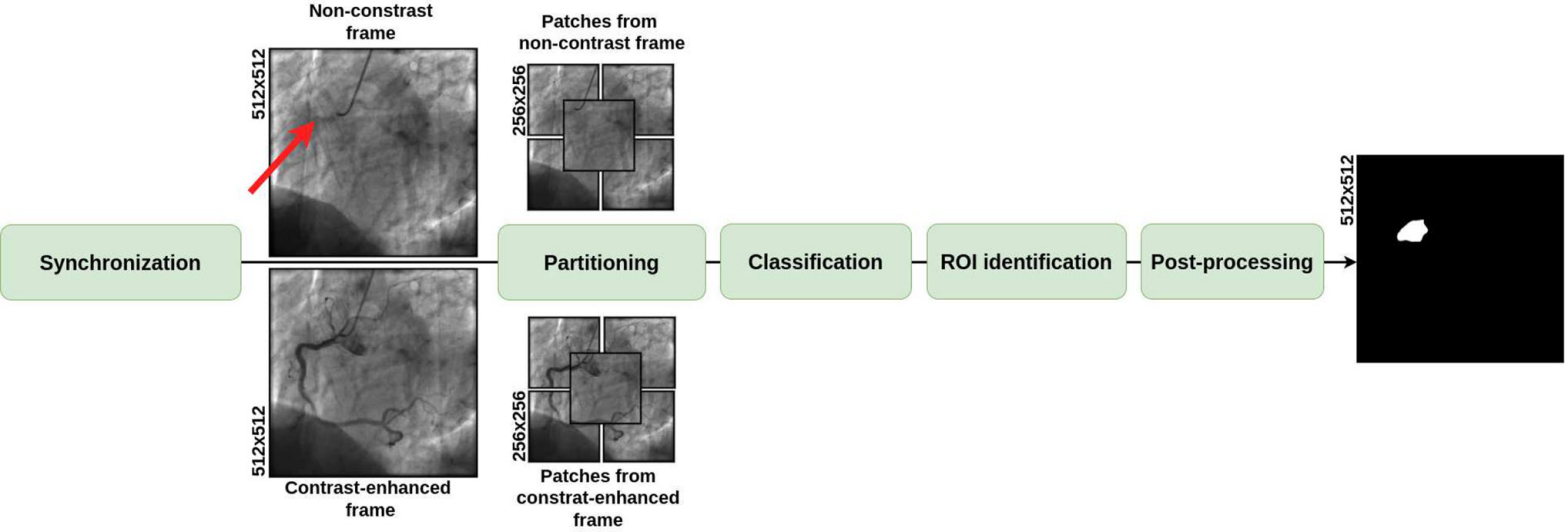
Overview of the entire workflow. The process begins with image frame synchronization, followed by partitioning each 2DCA frame into five 256$\times$256 patches. These patches are then classified one by one and used for ROI identification. A post-processing step is finally applied to refine the final output. The red arrows indicate the presence of CAC, annotated by physicians on the non-contrast frame

## Materials and methods

The following sections introduce the image dataset used in this study, the frame synchronization methods, and the multi-model DL network. An overview of the workflow is provided in Fig. 1.

### Dataset and annotations

The dataset consisted of 14 patients, each undergoing multiple 2DCA acquisitions with varying projection angles, for a total of 44 2DCA video sequences.^1^ The retrospective study was approved by the Ethics Review Board (Comitato Etico Regione Calabria—Area Centro): n. 137 25/05/2023 (CAL.HUB.BRIA). Each 2DCA acquisition included about 200 frames (15 fps), $512 \times 512$ pixels with a pixel size in the range of (0.20–0.39) mm. The first half of the video sequence was acquired without contrast and the remaining part using contrast. Three experienced cardiologists were invited to manually annotate CAC within 2DCA non-contrast images, guided by contrast-enhanced frames using the 3D Slicer Imaging Computing Platform [19]. The final ground truth segmentation was determined by majority voting among the three annotations, thereby mitigating possible annotation biases.

Each annotated non-contrast frame was paired with a contrast-enhanced frame (see Sect. 2.2) and given as input to our double-head classifier. Leave-one-out cross-validation (LOO-CV) was implemented to assess the effectiveness of the proposed methodology on variable data sets. For each fold, one acquisition was left as test set. The remaining acquisitions were used as training set.

### Image frame synchronization

**Fig. 2:**
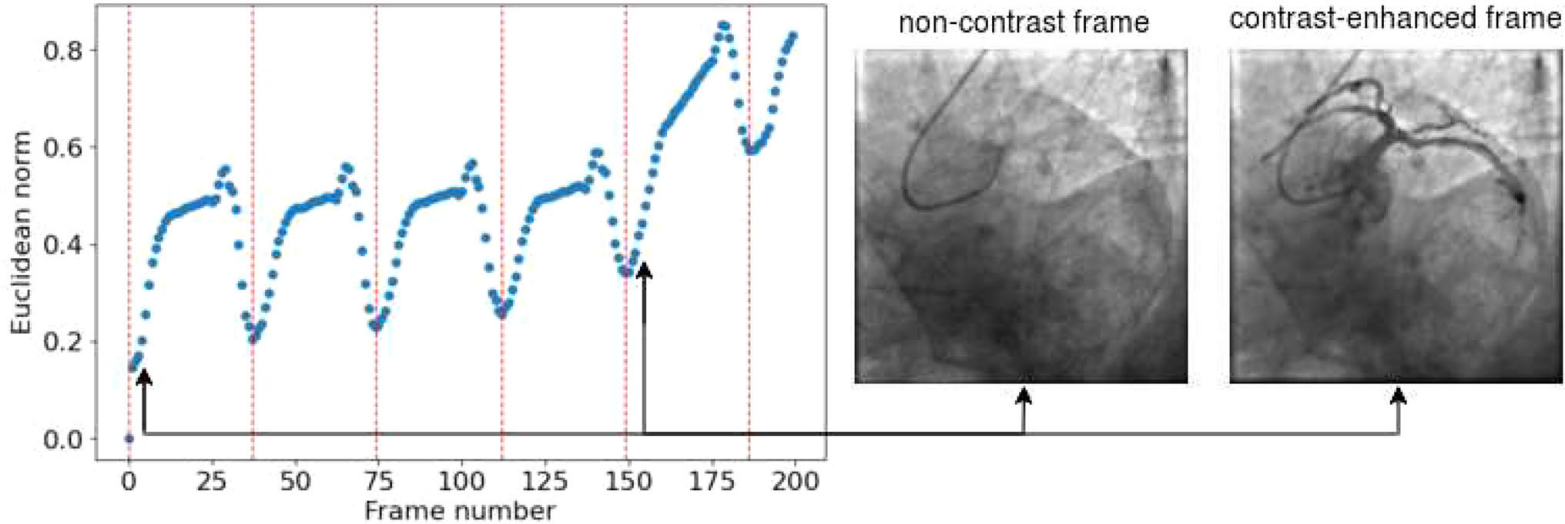
Example of the $S_k$ signal obtained by computing the Euclidean norm computed for a sample acquisition. It is evident that the signal manifests a distinct repetitive pattern. Once the FFT has been applied, it is possible to identify the period of the ECG-like signal. The red dashed lines mark the transition between one repetition and the subsequent. The corresponding frames are displayed on the right, with black arrows indicating the blue points in the signal

In principle, the electrocardiogram (ECG) signal could be used to synchronize contrast-enhanced and non-contrast (including the annotations) frames according to the heartbeat. However, it is usually not possible to retrieve the ECG and match its timestamp to the image acquisition, due to restrictions imposed by the equipment manufacturers. The frames synchronization is important because by comparing these frames and observing dynamic changes across the cardiac cycle, cardiologists can identify calcifications as persistent, static features aligned with anatomical structures. To overcome this limitation, we derived an ECG-like signal directly from the 2DCA acquisition. Using the first frame as a reference, we computed the Euclidean norm between the first frame and each of the subsequent frames, as it follows:

1 $$\begin{aligned} \text {dist}_{\text {norm}}(f_0, f_k) = \frac{\sqrt{\sum _{i=1}^{n} \sum _{j=1}^{m} (f_{0ij} - f_{kij})^2}}{\sqrt{\sum _{i=1}^{n} \sum _{j=1}^{m} f_{0ij}^2} + \sqrt{\sum _{i=1}^{n} \sum _{j=1}^{m} f_{kij}^2}} \end{aligned}$$

where $f_0$ is the first frame and $f_k$ is $k-th$ frame ($\forall k \in [1,N]$, with *N* = total number of frames) and $n \times m$ represents the spatial resolution of each frame. The sequence $dist_{\text {norm}}(f_0, f_k)$ represents a discrete signal $S_k$, consisting of N points (see Fig. 2). Fast Fourier transform was applied to $S_k$ to estimate the fundamental frequency and, as a consequence, the period of the ECG-like signal. Finally, non-contrast and contrast-enhanced frames were paired on the basis of the estimated period.

### Double-head ResUNet-18 architecture

**Fig. 3:**
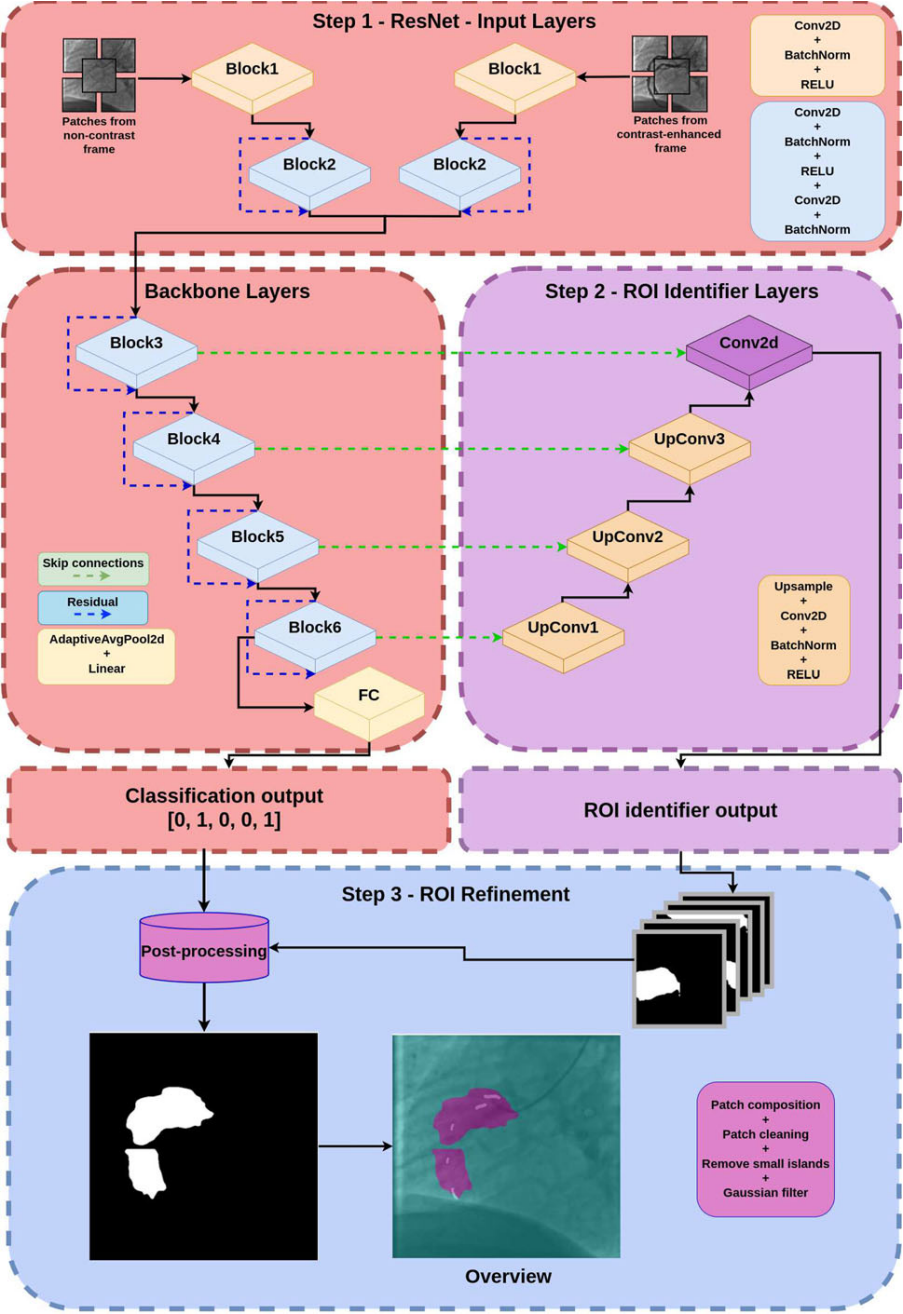
Detailed overview (with layer clarification) of the approach for classification, ROI identification, and ROI refinement. The outputs of the convolutional layers are employed to reconstruct and segment a ROI, while the classificator prediction is used to clean false positives pixels in the generated ROI. Finally, the predicted ROI is refined to obtain a compact area

**Fig. 4:**
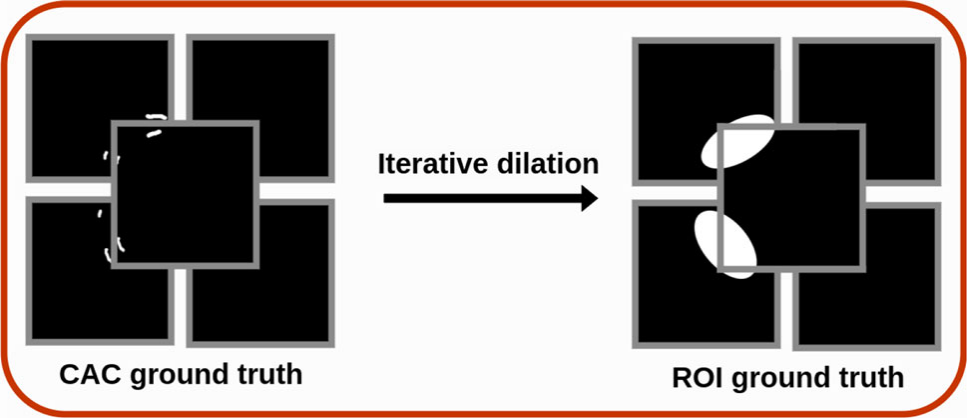
Generation of ${ROI}_{GT}$: different dilation steps were applied to the CAC annotations to define the target area, ${ROI}_{GT}$, in order to train the decoder branch

We propose a multi-model architecture that combines a double-head ResNet-18 [20] backbone (a residual network known for its classification ability) with a U-Net style decoder to perform the identification of regions containing CAC. Referring to Fig. 1, the first step (Step 1) allows to classify the images and to extract information that will be used as skip connections in the second step (Step 2) using the double-head ResNet-18. The information obtained from the Step 2 are employed to identify the ROI with the U-Net style decoder. More in detail:

- The ResNet-18 [20] serves as the encoder, or feature extractor. Its residual blocks help in learning hierarchical representations, especially useful in classification tasks. Each image frame is partitioned into five overlapping patches of $256 \times 256$ pixels, which are used as input, as shown in Fig. 1. This approach reduces the image size while preserving fine details, in order to focus the model on small-scale information associated with the location of the CACs, while maintaining the global information derived from different points in the image itself. Also, this approach introduces data augmentation to reduce bias.
  This part of the architecture can be divided into two sub-architectures: head layers and tail layers (Step 1, see Fig. 3). The head layers accept as input the synchronized image pair. Each head consists of the first block (Block1), composed by a 2D convolution, batch normalization and ReLU, and the second block (Block2), composed by a double 2D convolution, batch normalization, and ReLU with residual connection. The dual input provided to the network was used in Step 1 to maximize feature extraction: the non-contrast frame was used to identify subtle indicators of CAC’s, such as texture differences, shadowing effects, or localized brightness, while the contrast-enhanced was employed to provide clear anatomical context, highlighting the position of vessels and showing luminal irregularities [2].
  The outputs of the two heads are concatenated and passed as input to the tail layers. The latter consist of four identical blocks, each containing two 2D convolution layers, a batch normalization layer, and a ReLU activation layer with residual connections, followed by a fully connected layer for classification.
- The U-Net-like decoder structure adds decoding layers that upsample the features learned by the double-head ResNet-18, allowing for spatially detailed predictions (in this specific case, the identification of ROIs in image frames). As shown in Fig. 4, the target ROI, referred to as the ground truth (${ROI}_{GT}$), was obtained by applying iterative dilation to the segmented CACs, and was used as the reference for the training.
  As shown in Fig. 3, the classifier’s prediction (presence or absence of calcification) helps reducing false positives in the identified regions. This step is crucial for improving the accuracy of the ROI, as it ensures that regions without CACs, as determined by the classifier, are excluded from the final ROI. Note that to ensure robustness and reliability, the classifier during this training is frozen.

## Experimental details

The implementation of the proposed workflow, including the LOO-CV, was carried out in Python, using the PyTorch framework.^2^ Fold data splitting was kept the same for both the classification with ResNet-18 backbone and the ROI identification via the U-Net-like decoder described in Fig. 3. For each fold, the classification model was trained using the Adam optimizer and the binary cross-entropy loss, with a learning rate set to $10^{-4}$ and a batch size of 32 for 100 epochs. At the end of each classification epoch, the model’s performance was evaluated on the validation set using the F1-score and the explanation from the Grad-CAM [21] output, extracted from the last block (Block6, see Fig. 3) to assess whether the classification step not only detected the presence of CAC but also determined whether the model focused on the correct areas associated with the presence of CAC. The Grad-CAM outputs, which provided insight into the classifier’s predictions, are presented in Fig. 1 in the supplementary material.

Additionally, the ROI identification model was optimized using the Adam optimizer with a learning rate of $10^{-3}$ and a batch size of 32 for 100 epochs. In order to provide feedback to the ROI identification model, the boundary loss function [22] was employed.

A sensitivity study was conducted to investigate on the impact of $ROI_{GT}$ dilation on the network performances. The number of iterations was changed while keeping the hyperparameters fixed, with each iteration extending the ROI by five pixels following an elliptical shape. This experiment was conducted on 50% of the entire dataset in order to limit the required computational and time resources. The optimal number of iterations was then used to perform the final experiment on the whole dataset. The performance of ROI identification model (U-Net-like decoder) was evaluated at the end of each epoch on the validation set using intersection over minimum (IoM) metric [23] to quantify how well the extracted ROI overlaps with the CAC and Hausdorff distance (HD) [24] to measure the surface distance between the ROI and the CAC.

In reference to Fig. 3 (Step 3—ROI refinement), small islands were removed from the identified ROI using the scikit-image [25] $remove\_small\_object$ method with a $min\_size = 1000$. A Gaussian filter was then applied, with a kernel standard deviation of $\sigma$ varying between 1, 3, and 5. Several experiments were conducted and statistically validated to assess the impact of post-processing. The results of these experiments are reported in Table 1 of the supplementary material.

Furthermore, a study was conducted to assess the feasibility of detecting CACs or ROI with YOLO, as proposed by Osama et al. in [11] for the recognition of blockages in coronary angiographic images and other state-of-the-art models such as U-Net, TransUNet, and Mask R-CNN, for the direct segmentation of the ROI.

## Results and discussion

**Table 1:** Results of the sensitivity study on the impact of CACs dilation (iterations) for the ${ROI}_{GT}$ generation and the convergency of the boundary loss in the U-Net branch. The IoM and HD metrics are shown for each experiment, with median and quartile values reported. The experiments were performed on a randomly selected 50% subset of entire the dataset, with the best-performing configuration (four iterations) applied to the final experiment on the full dataset

Experiment	Dataset size	Iterations	IoM (median ± quartiles)	HD [mm] (median ± quartiles)
1	50%	0	0.03 (0.0$-$0.17)	184.40 (95.31$-$217.22)
2	50%	3	0.65 (0.45$-$0.78)	50.99 (40.98$-$59.83)
3	50%	4	0.69 (0.55$-$0.85)	**47**.**93 (36**.**71** $-$**60**.**17)**
4	50%	6	**0**.**75 (0**.**53** $-$**0**.**90)**	51.77 (40.06 - 68.12)
Final	100%	4	**0**.**64 (0**.**46** $-$**0**.**86)**	**47**.**16 (35**.**05** $-$**63**.**77)**

As illustrated in Table 1, a total of four iterations to dilate the CACs and create the target ROI for the identification (Experiment 3) represent the optimal configuration for balancing the IoM and Hausdorff distance scores, effectively incorporating CACs while minimizing the ROI dimension.

The results obtained with the proposed double-headed ResUNet-18 architecture and a kernel standard deviation of $\sigma =3$ for the post-processing Gaussian filter are shown in Table 2. The ablation study performed to assess the impact of the post-processing and in particular of the $\sigma$ value, was reported and discussed in the supplementary material (see "Post-processing ablation study and statistical analysis").

**Fig. 5:**
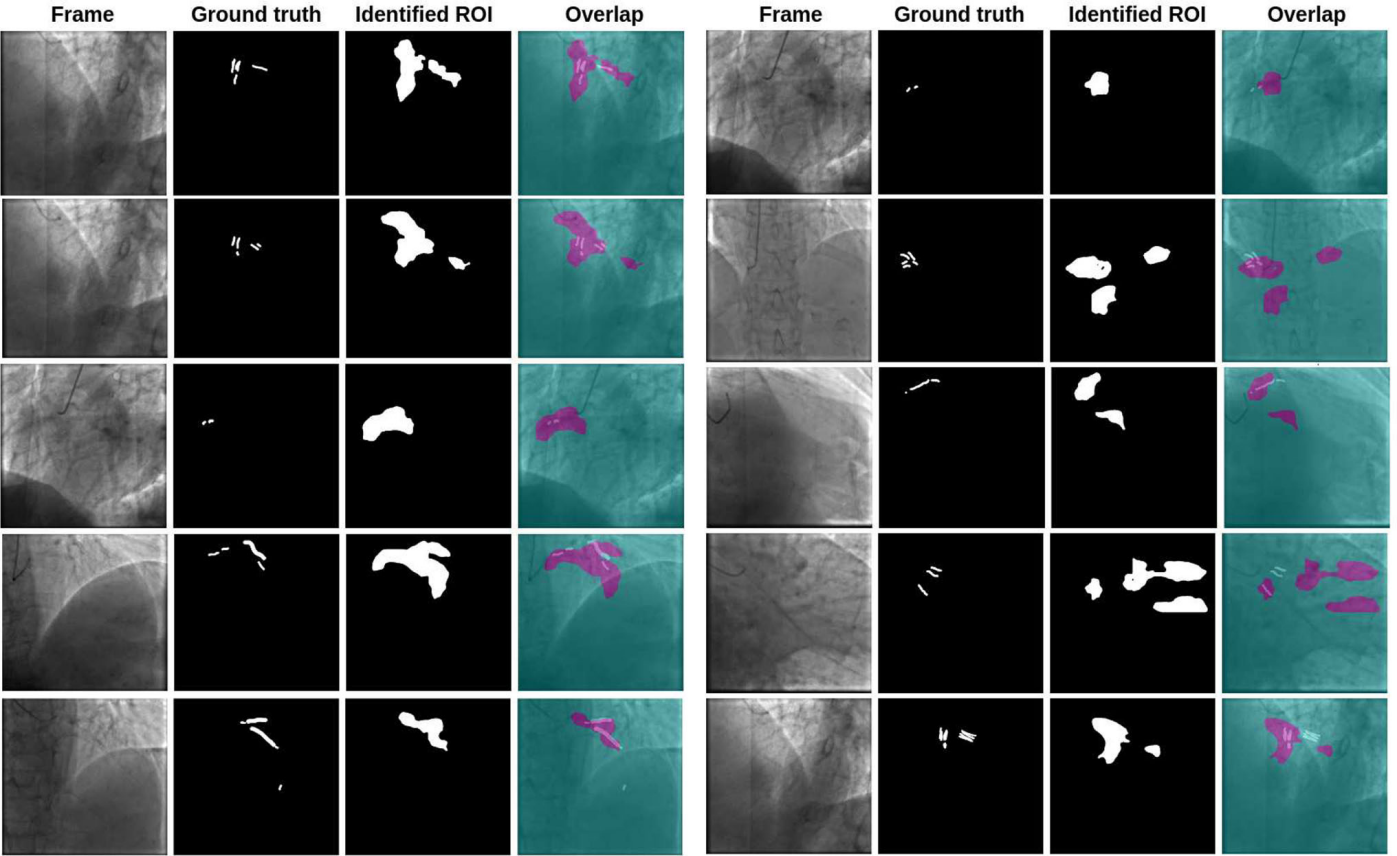
A grid comparison is presented between the 2DCA frame, the corresponding annotated CAC, and the region identified by the model. Additionally, an overlap of the images is provided. On the left, examples of the best cases are illustrated, while on the right, examples of the worst cases are presented

The results reported in Table 2 demonstrate that the classification model can accurately identify positive instances, with a precision ($\text {median} \pm \text {quartiles}$) of 0.89 (0.81$-$0.94) and a comparable recall ($\text {median} \pm \text {quartiles}$) of 0.89 (0.79$-$0.95). This balance between precision and recall is also reflected in the F1-score ($\text {median} \pm \text {quartiles}$) of 0.87 (0.77$-$0.94), which indicates an overall satisfactory performance. In the identification task, which in this case consists of identifying a ROI that encloses the segmented ground truth CACs, we obtained an IoM ($\text {median} \pm \text {quartiles}$) of 0.64 (0.46$-$0.86). The Hausdorff distance, employed to quantify the area distance between the ROI and the CACs, has a value ($\text {median} \pm \text {quartiles}$) of 47.16 (35.05$-$63.77) mm. The observed variability indicates that, in some instances, false positive ROIs may be generated (see Fig. 5). Classification performance contributed to improved ROI identification, as the patches were refined using the classifier.

**Table 2:** Performances of the classification and identification models in the proposed double-head ResU-Net18. Classification metrics include precision, recall, and F1-score, while identification metrics include intersection over minimum (IoM) and Hausdorff distance (HD). Results of different folds are presented as median ± quartiles

**Classification**		
	Precision (median ± quartiles)	0.89 (0.81$-$0.94)
	Recall (median ± quartiles)	0.89 (0.79$-$0.95)
	F1-score (median ± quartiles)	0.87 (0.77$-$0.94)
Identification		
	IoM (median ± quartiles)	0.64 (0.46$-$0.86)
	HD (mm) (median ± quartiles)	47.16 (35.05$-$63.77)

In Fig. 5, we illustrate the results of the complete workflow. The image shows, for each case, the original frame, CAC ground truth, the identified ROI, and the overlap between the original frame, CAC ground truth, and predicted ROI. On the left, we show the best-performing cases, while on the right, the worst-performing cases are presented. As can be observed, the identified ROIs consistently capture at least part of the CAC ground truth. Although the predicted ROIs are not particularly large, they are located in specific, consistent areas of the frame.

**Fig. 6:**
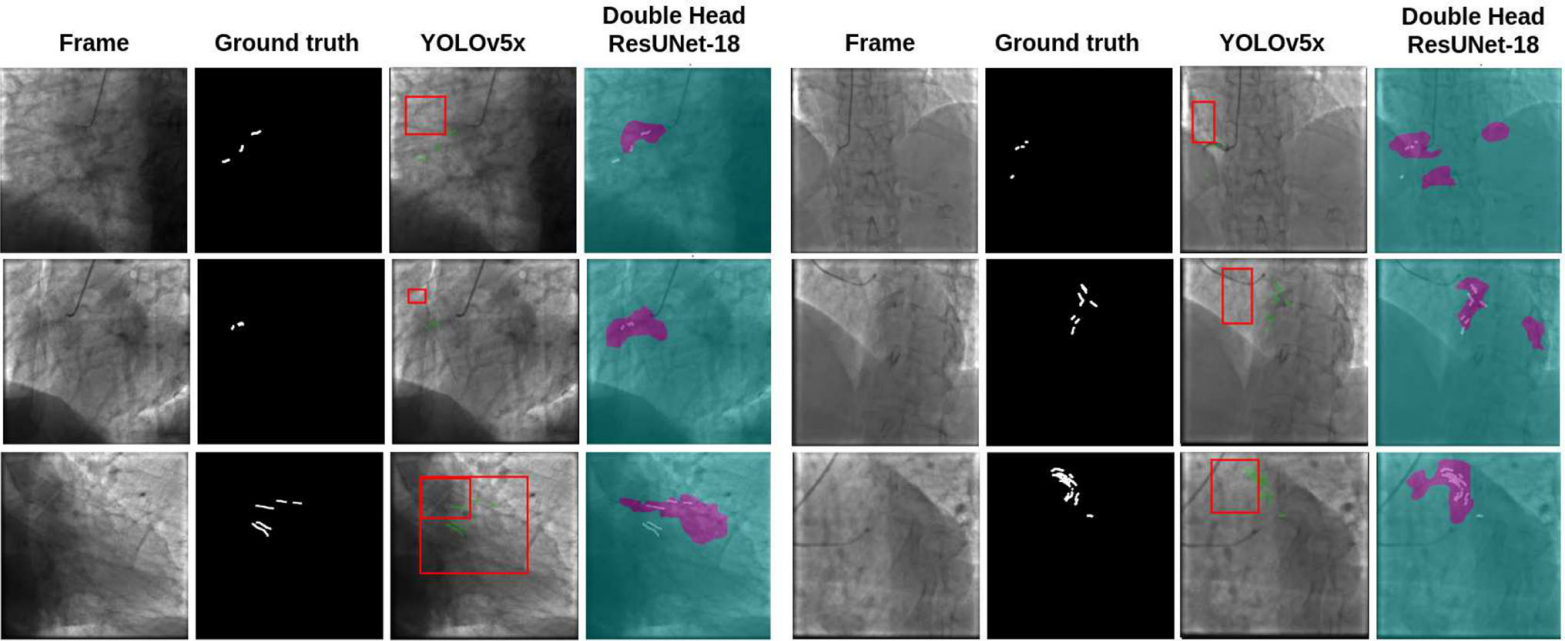
The image presents a grid comparison between our double-head ResUNet-18 model and the best-performing YOLOv5x model for CAC identification. In the comparison, the YOLOv5x model’s output bounding boxes are shown in red, while the ground truth boxes are in green. The corresponding ROI identified by our model is highlighted in pink, with the ground truth ROI displayed in white. This visual comparison highlights the differences in ROI detection performance between the two models

As may be observed in Fig. 5 (column 2, row 1), the identified ROI may approach but not fully enclose the CACs when these are very small. In other instances of failure, such as those illustrated on the right in Fig. 5, false positive ROIs may be identified when the CACs are not distributed evenly or are distant from one another.

As shown in Fig. 6, the YOLO framework has been observed to have difficulty in detecting small objects within complex images, such as those exhibiting low contrast. The detection of small objects, such as CAC, is frequently unsuccessful or inaccurate when they do not occupy the majority of the detection grid. Even the creation of larger bounding boxes did not result in a reduction of false positives or an improvement in the identification of fine details. We also conducted further experiments for direct ROI segmentation by using U-Net [12], TransUNet [26], and Mask R-CNN [27] architectures (see Table 2 in the supplementary material). Our approach, which employs a classification branch to direct the identification of ROIs, resulted superior (*p* values < 0.05, Wilcoxon test).

## Conclusion

Despite the intrinsic complexity of the application, particularly due to the low contrast within the images and the small size of the target, we developed a modular and specific workflow that improves the identification of CAC using 2DCA. To the best of our knowledge, this is the first study to make such an attempt. This workflow integrates two different steps, each of which contributes to the overall improvement of the ROI identification process. Inspired by the clinical method, the modular design enables targeted optimizations, refining the identification process. Moreover, each step can be used independently, adding versatility in clinical or research settings. Future work could aim for fully automated segmentation, taking into account the time-dependent nature of the acquisitions.

## Supplementary Information

Below is the link to the electronic supplementary material.

Supplementary file 1 (pdf 446 KB)

## Footnotes

1 Acquired using the X-Ray Angiographic SOP, DLX Series 01 from GE HealthCare, at the Department of Experimental and Clinical Medicine, Magna Graecia University.

2 To train the models, we used an NVIDIA A100 80GB GPU, 16 CPU cores, and 64GB of RAM from a Supermicro AS-4124GS-TNR 4U Rackmount GPU SuperServer.
