# Supplementary material for: A multi-model deep learning approach for the identification of coronary artery calcifications within 2D coronary angiography images
Source: Int J Comput Assist Radiol Surg. 2025 May 8;20(6):1273–81. doi: 10.1007/s11548-025-03382-5 (PMC12167256; doi:10.1007/s11548-025-03382-5)
Supplement: Supplementary file 1 — (pdf 446 KB) [file 11548_2025_3382_MOESM1_ESM.pdf]

A Multi-Model Deep Learning Approach for the Identification of Coronary Artery Calcifications within 2D Coronary Angiography Images

Edoardo De Rose[1*†], Ciro Benito Raggio[2*†], Ahmad Riccardo Rasheed[4], Pierangela Bruno[2], Paolo Zaffino[3], Salvatore De Rosa[**4], Francesco Calimeri[**2,5], Maria Francesca Spadea[**2]

[1]Department of Mathematics and Computer Science, University of Calabria, Pietro Bucci, Rende, 87036, Calabria, Italy.
[2]Institute of Biomedical Engineering, Karlsruhe Institute of Technology, Fritz-Haber-Weg 1, Karlsruhe, 76131, Baden-Württemberg, Germany.
[3]Experimental and Clinical Medicine, Magna Graecia University, Viale Europa, Catanzaro, 88100, Calabria, Italy.
[4]Medical and Surgical Sciences, Magna Graecia University, Viale Europa, Catanzaro, 88100, Calabria, Italy.
[5]DLVSystem Srl, Viale della Resistenza 19/C, Rende, 87036, Calabria, Italy.

*Corresponding author(s). E-mail(s): edoardo.derose@unical.it; ciro.raggio@kit.edu;
Contributing authors: ahmadriccardo.rasheed@studenti.unicz.it; pierangela.bruno@unical.it; p.zaffino@unicz.it; saderosa@unicz.it; francesco.calimeri@unical.it; mf.spadea@kit.edu;
†These authors contributed equally to this work.

[1**] These authors share senior authorship.

Supplementary material

Grad-CAM Explanation

At the end of each classification epoch, model's capabilities were evaluated also using the Grad-CAM output.

As shown in Figure I, Grad-CAM-generated heatmaps demonstrate the regions to which the model directed its attention during the classification process, thereby influencing the final prediction. It can be observed that, in case of positive prediction (presence of CACs, shown on the left side of the Figure I), the model primarily identifies as significant the areas corresponding to the ground truth CACs.

This evaluation revealed that the model focuses on the correct regions associated with the presence of CAC in cases where a positive prediction was made.

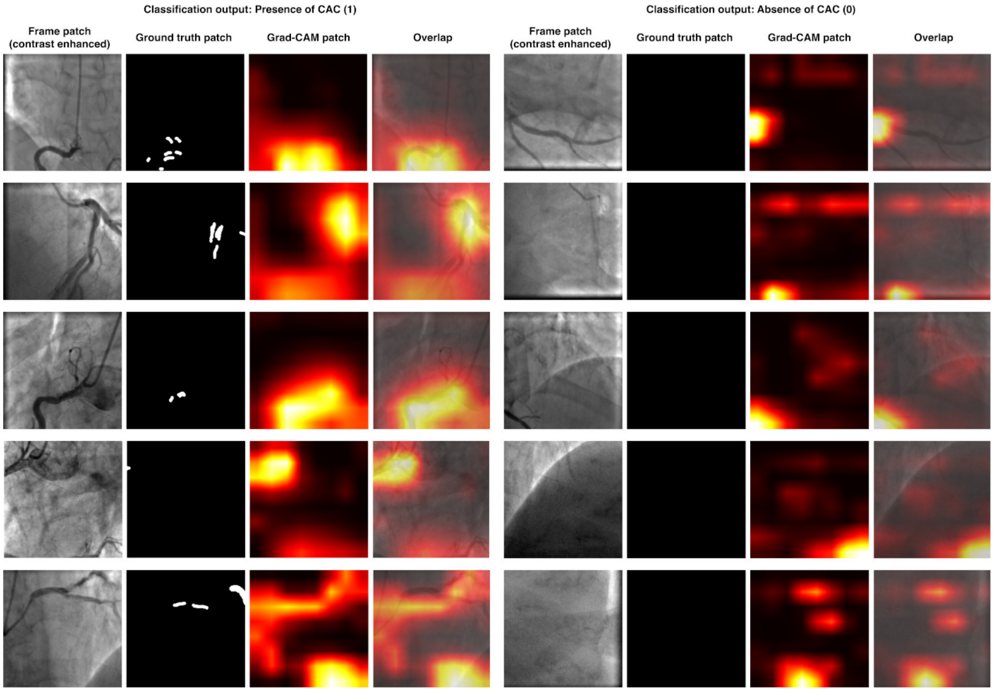

Fig. I Grad-CAM output visualization. The left grid highlights cases where the model correctly identified the presence of CACs (class 1), while the right grid shows examples of predictions for the absence of CACs (class 0), showing the relationship between the model's classification and the regions it identified as significant for its decision-making process. In case of positive predictions (left grid), the Grad-CAM output aligns closely to the areas corresponding to the ground truth CACs, highlighting the model's focus on relevant regions.

Post-processing ablation study and statistical analysis

To assess the influence of post-processing on the raw predictions, several experiments were conducted. Each experiment applied the post-processing with different strategies and values to the model's raw predictions, and the results were compared in terms of Intersection over Mask (IoM) and Hausdorff Distance (HD). The experiments are summarized as follows:

- **Experiment 1:** No post-processing. The raw predictions from the model were directly evaluated.
- **Experiment 2:** Application of a Gaussian filter with a kernel standard deviation of $\sigma = 3$ to smooth the predictions.
- **Experiment 3:** Combination of small island removal and a Gaussian filter with $\sigma = 1$ to enhance predictions.
- **Experiment 4:** Application of small island removal followed by a Gaussian filter with $\sigma = 3$.
- **Experiment 5:** Application of small island removal followed by a Gaussian filter with $\sigma = 5$.

The performance metrics for each experiment are reported in Table I.

Table I Performance metrics for the evaluation of post-processing strategies.

Experiment	IoM (median ± quartiles)	HD [mm] (median ± quartiles)
1	0.65 (0.46-0.86)	57.52 (44.37-68.44)
2	0.65 (0.46-0.86)	55.56 (42.13-68.19)
3	0.64 (0.46-0.82)	46.64 (35.31-57.71)
4	0.64 (0.46-0.86)	47.16 (35.05-63.77)
5	0.64 (0.46-0.86)	46.39 (34.06-63.31)

An analysis of the results indicates that the application of the remove small island combined with a Gaussian filter as a post-processing method (Experiments 3, 4 and 5) is the optimal approach to preserve the ROI overlap on the CACs as expressed by the IoM metric while obtaining a median enhancement of 10mm in terms of HD (see Experiment 3, 4 and 5 in Table I). This is reflected in a more constrained and precise ROI, compared to the experiment conducted without the application of post-processing (Experiment 1).

The Kruskal-Wallis H-test was performed to assess that the ROI overlap (IoM) is not significantly impacted by post-processing, obtaining a $p - value = 0.99$, thus confirming the null hypothesis.

Furthermore, the Wilcoxon paired test was performed to confirm that the application of Experiment 3, 4 and 5 post-processing can significantly improve the result compared with the absence of post-processing (Experiment 1) in terms of HD. This was determined by obtaining a $p - value = 4 \times 10^{-4}$ when $\sigma = 1$, a $p - value = 3.49 \times 10^{-7}$ for $\sigma = 3$, and a $p - value = 1.77 \times 10^{-7}$ when $\sigma = 5$, thus

confirming the significant improvement in terms of HD when the Experiment 3, 4 and 5 post-processing methods were applied.

Therefore, the Kruskal-Wallis H-test was conducted to evaluate the statistical significance of the σ value for the application of the Gaussian filter. The analysis yielded a $p - value = 0.96$ when σ assumed the values 1, 3, and 5, indicating the absence of statistically significant differences.

Consequently, it can be concluded that the removal of small islands combined with the Gaussian filter enables the narrowing of the ROI on the CACs, thereby minimizing the HD metric while preserving the overlap as expressed through the IoM.

Models ablation study and statistical analysis

To validate the efficacy of the proposed Double-head ResUNet-18 model, further experiments were conducted to enable the direct segmentation of the region of interest (ROI), and the result for each experiment is reported in Table II. The selected models are founded on the related works and also on the logic of their implementation. In addition to YOLO, the simple UNet underlying the proposed model was evaluated, as well as the TransUNet architecture to incorporate the logic of modern transformers, and finally the MaskRCNN using the ResNet architecture as a backbone.

Table II Results of the models ablation study.

**Model**	**IoM** (median ± quartiles)	**HD [mm]** (median ± quartiles)
MaskRCNN (w/ResNet backbone)	0.03 (0.0-0.19)	171.29 (111.35-209.74)
TransUNet	1.0 (1.0-1.0)	140.05 (127.89-146.82)
UNet	0.72 (0.46-0.86)	66.07 (51.66-79.66)
Double-head ResUNet-18 **(our)**	0.64 (0.46-0.86)	47.16 (35.05-63.77)

The UNet and TransUNet models receive a multi-channel input of dimensions 256×256 comprising contrast-enhanced and non-contrast frames, employing the identical settings used to train the proposed Double-head ResUNet-18 model.

The MaskRCNN model receives in input a multi-channel (contrast-enhanced and non-contrast full image) images of size 512×512, as the original MaskRCNN framework necessitates the incorporation of at least one label within each image. Consequently, it was necessary to pass the entire image to ensure the presence of CAC in all samples.

The cross-validation method employed was identical to the one used for the proposed approach (LOO-CV), using the same folds. The evaluation process was performed using the same post-processing pipeline of the Double-head ResUNet-18

to enhance the final outcome.

The Mask R-CNN architecture was unable to segment the ROI, as shown by the performance metrics reported in Table II: low IoM indicates minimal overlap with the true regions, while high Hausdorff distance highlights high spatial distance between the predicted ROI and the ground truth CACs.

The TransUNet architecture, despite integrating transformer components, failed to learn the task. Its outputs across the test dataset were uniform white squares. The maximum IoM (see Table II) is thus obtained because of the complete overlap of the square on the CACs. In fact, this is confirmed by a high Hausdorff distance.

The UNet architecture, a foundation for our Double-head ResUNet-18 model, demonstrated the ability to segment the ROI but performed worse than our approach. While UNet achieved higher IoM, its larger Hausdorff distance revealed less precise predictions compared to the proposed model, due to the larger area predicted as the ROI by this model. This finding underscores the efficacy of the proposed model in enhancing the UNet standard and outperform other logic and architectures for this task.

We performed Wilcoxon paired tests to assess the statistical significance of our model comparisons, with p-values such as $5,2 \times 10^{-8}$ for the Double-head ResUNet-18 vs. MaskRCNN, $1,9 \times 10^{-8}$ for the Double-head ResUNet-18 vs. TransUNet and 8×10^{-4} for the Double-head ResUNet-18 vs. UNet indicating significant differences.
